# Prognostic value of radiation interruption in different periods for nasopharyngeal carcinoma patients in the intensity‐modulated radiation therapy era

**DOI:** 10.1002/cam4.3580

**Published:** 2020-10-27

**Authors:** Xing‐Li Yang, Guan‐Qun Zhou, Li Lin, Lu‐Lu Zhang, Fo‐Ping Chen, Jia‐Wei Lv, Jia Kou, Dan‐Wan Wen, Jun Ma, Ying Sun, Yan‐Ping Mao

**Affiliations:** ^1^ Department of Radiation Oncology State Key Laboratory of Oncology in South China Guangdong Key Laboratory of Nasopharyngeal Carcinoma Diagnosis and Therapy Sun Yat‐Sen University Cancer Center Collaborative Innovation Center for Cancer Medicine Guangzhou People’s Republic of China; ^2^ Department of Molecular Diagnostics State Key Laboratory of Oncology in South China Guangdong Key Laboratory of Nasopharyngeal Carcinoma Diagnosis and Therapy Sun Yat‐sen University Cancer Center Collaborative Innovation Center for Cancer Medicine Guangzhou People’s Republic of China

**Keywords:** chemotherapy, interruption, nasopharyngeal carcinoma, radiation therapy, survival

## Abstract

We aimed to investigate the prognostic value of radiation interruptions at different times on the overall survival (OS) and disease‐free survival (DFS) of patients with nasopharyngeal carcinoma receiving intensity‐modulated radiation therapy. Totally, 4510 patients were identified from a well‐established big‐data intelligence platform. Optimal interruption thresholds were identified using Recursive partitioning analyses. Actuarial rates were plotted using the Kaplan–Meier method and were compared using the log‐rank test. Patients with preceding interruptions ≥1 d (5‐year OS, 89.6% vs. 85.7%, *p* < 0.001; 5‐year DFS, 81.4% vs. 76.4%, *p* < 0.001), or latter interruptions ≥4 d (88.4% vs. 82.3%, *p* < 0.001; 79.2% vs. 75.1%, *p* = 0.006) showed significant detrimental effects on OS and DFS than patients without those interruptions. However, no significant lower survival was identified in latter interruptions ≥1 d (5‐year OS: 89.0% vs. 86.7%, *p* = 0.053; 5‐year DFS, 80.2% vs. 77.8%, *p* = 0.080). Latter interruptions ≥4 d was an independent unfavorable prognostic factor for OS (HR, 1.404; 95% CI, 1.143–1.723, *p* = 0.001) and DFS (HR, 1.351; 95% CI, 1.105–1.652, *p* = 0.003) in multivariate analysis. Radiation interruptions longer than 3 days that occurred in the latter period of treatment with IMRT were independent factors in poorer survival. Efforts are needed to minimize radiation interruptions and improve the timely provision of treatment.

## Graphical abstract

Our findings suggest that radiation interruptions longer than 3 days that occurred in the latter period of treatment with IMRT were independent factors in poorer survival. Efforts are needed to minimize radiation interruptions and improve the timely provision of treatment.

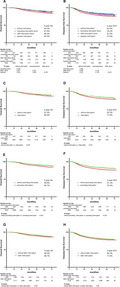

## 1 INTRODUCTION

Nasopharyngeal carcinoma (NPC) is an uncommon malignancy worldwide but endemic in Southeast Asia, especially China, with an estimated 42,100 new cases and 21,320 deaths in 2013.^1^, ^2^

The primary treatment option for nondisseminated NPC is radical radiotherapy, which should be given without interruption. The current radiation protocol for NPC, which consists of five fractions per week uninterrupted, for more than 6 weeks, is mainly based on a large‐scale study of head and neck cancer by a Danish research group.^3^ Unfortunately, NPC was not included in that study. Radiotherapy interruptions, which are usually induced by machine malfunctions, limited medical resources, holidays, and severe acute treatment‐related toxicity, are believed to increase the risk of treatment failure due to the repopulation of tumor cells.^4^, ^5^ Prolonged radiotherapy treatment has been found association with poorer prognoses among patients with NPC treated by two‐dimensional radiation therapy (2DRT).^6^

Currently, intensity‐modulated radiation therapy (IMRT) is more commonly used than 2DRT or three‐dimensional radiation therapy (3DRT) to treat NPC, as IMRT can provide better tumor control while decrease incidence of radiation‐related toxicities.^7^, ^8^, ^9^ However, the role of radiation interruption has remains controversial for IMRT. Disagreement about this center on whether the interruption is associated with inferior survival outcomes and the length of the interruption necessary to impair survival.^10^, ^11^, ^12^, ^13^, ^14^, ^15^

The studies above have concentrated on the overall radiotherapy treatment time or interruption of the entire radiation treatment; however, the time of the interruption might have also led to different results. Interruptions generally occurred at the beginning or toward the end of treatment with the use of 2DRT, and failed to be a significant prognostic index in subgroups of patients with NPC, who experienced an interruption.^6^ The above results may have changed because of the improved management. However, no study has focused on the times of interruptions in IMRT. Therefore, we designed a study to investigate the value of the occurrence of interruptions at different times during IMRT on overall survival (OS) and disease‐free survival (DFS) of patients with NPC.

## 2 METHODS AND MATERIALS

### 2.1 Patient selection

This retrospective study was conducted using 4510 patients with histologically proven, nonmetastatic NPC, treated with IMRT between January 2013 and December 2015 in the authors’ center. Patients failed to finish planed radiotherapy were excluded. The Clinical parameters were retrieved from the big‐data intelligence platform. Before treatment, every patient received evaluation included complete patient history, general physical examination, contrast‐enhanced magnetic resonance imaging (MRI) of the nasopharynx and neck, fiberoptic nasopharyngoscopy, abdominal ultrasonography or computed tomography (CT), whole‐body bone scan, and blood profile. Positron emission tomography and CT (PET‐CT) would be recommended for patients with suspected metastasis. Each patient was restaged using the eighth edition of the American Joint Commission on Cancer staging system.^16^

### 2.2 Treatment

Patients enrolled were treated with radical IMRT daily for 5 days per week delivered for 6–7 weeks. The prescribed doses for planning target volume (PTV) of the primary gross tumor volume (GTVnx) were 66–72 Gy/28–35 fractions, the GTV in the involved lymph nodes were (GTVnd) 64–70 Gy/28–35 fractions, the high‐risk clinical target volume (CTV1) were 60–63 Gy/28–35 fractions and the low‐risk clinical target volume (CTV2) were 54–56 Gy/28–35 fractions.

Overall, 83.2% (3752/4510) of the patients received platinum‐based chemotherapy. About 62.3% (2258/3619) CCRT consisted of cisplatin, were delivered weekly (30–40 mg/m^2^) or on weeks 1, 4, and 7 (80–100 mg/m^2^) of radiotherapy (Table S1). The induction chemotherapy (IC) or adjuvant chemotherapy (AC) consisted of cisplatin (75 mg/m^2^ d1) with docetaxel (75 mg/m^2^ d1), cisplatin (75 mg/m^2^ d1) with 5‐fluorouracil (1000 mg/m^2^ d1–d5), or cisplatin (75 mg/m^2^ d1) with 5‐fluorouracil (1000 mg/m^2^ d1–d5), and docetaxel (75 mg/m^2^ d1) every 3 weeks for two to four cycles. Some patients received AC with oral capecitabine. (Table S2).

### 2.3 Definitions of interruptions during the different phases of treatment

Overall treatment time was calculated as the duration of radiotherapy (from the first day to last day of the planned treatment course). All patients were treated with no planned interruptions. Interruption was defined as the overall treatment time minus the planned radiation time. Median fraction was calculated as rounded “N/2” (assuming “N” was the planned course). The planned date of the median fraction was defined as the date the median fraction was received without interruption. The duration of the preceding interruption (interruption occurred before the median fraction) was defined as the actual date of the median fraction minus the planned date of the median fraction. The duration of the latter interruption (interruption occurred after the median fraction) was defined as duration of the interruption minus the duration of the preceding interruption.

The cohort was classified as the (a) without interruption and interruption groups, according to whether patients experienced interruptions; (b) without preceding interruption and preceding interruption groups, according to whether patients experienced preceding interruptions; and (c) without latter interruption and latter interruption groups, according to whether the patients experienced latter interruptions.

### 2.4 Follow‐up

During the first 3 years after treatment, patients returned for follow‐up examination every 3–6 months and every 6–12 months from the fourth year until death. Telecommunication would be the supplementary mean if their recent follow‐up examinations were not recorded in medical records. The primary end point was OS, calculated as the time from the date of initiate treatment to the date of death or the last follow‐up visit. The secondary end point was DFS, calculated as the time from the date of initiate treatment to the date of first relapse at any site, death or the last follow‐up visit. The median follow‐up was 56.6 months (range = 2.1–76.6 months).

### 2.5 Statistical analysis

The host factors included gender (male vs. female), age (<45 years vs. ≥45 years), smoking status (no vs. yes), drinking status (no vs. yes), family history of cancer (no vs. yes), and whether the carcinoma has concomitant (no vs. yes). Tumor factors consisted of pathological types (WHO type I–II vs. WHO type III), T stage (T1–2 vs. T3–4), N stage (N0–1 vs. N2–3), and overall stage (I–II vs. III–IV). Treatment factors were prescribed fractions (28–31 f vs. 32–35 f), chemotherapy (no vs. yes), IC (no vs. yes), CCRT (no vs. yes), AC (no vs. yes), preceding interruption (<1 d vs. ≥1 d), latter interruption (< 1 d vs. ≥1 d), latter interruption (<4 d vs. ≥4 d), interruption (no vs. yes, 1 d), and interruption (no vs. yes, 1–4 d). The covariates between the groups were compared using Fisher's exact test or *χ* ^2^ test.

Optimal interruption thresholds were identified using Recursive partitioning analyses (RPAs). All the optimal thresholds (preceding or latter interruption) were calculated with the entire group. Time‐to‐event end points were plotted using the Kaplan–Meier method and were compared using the log‐rank test. The independent statistical significance of prognostic factors and hazard ratio (HR) were estimated using the Cox proportional hazards model. The proportional hazards assumption was graphically verified based on the Schoenfeld residuals.^17^

Propensity scores matching (PSM) were used to adjust the following variables: gender, age, smoking history, concomitant, T stage, N stage, overall stage, prescribed fractions, and preceding interruptions to create a well‐balanced cohort of latter interruption.

All analyses were performed with the rms package in R version 3.3.2 (http://www.r‐project.org/). *p* values were based on two‐sided tests, and *a* = 0.05 was the criterion for statistical significance.

## 3 RESULTS

### 3.1 Clinical characteristics

Approximately, 21.0% (946/4510) of the patients experienced treatment failure and 12.0%(541/4510） died during the follow‐up examination. Clinical characteristics are summarized in Table 1. It is more possible for patients with advanced T stage and overall stage NPC were to have interruption ≥1 d (*p* < 0.001 for all). Patients who received prescribed fractions of 32–35 and CCRT were more likely to experience preceding interruptions (*p* < 0.001 for all). (Table 1).

**TABLE 1:** Comparison of basic characteristics in the entire cohort and propensity score‐matched cohort

	Entire Group										PSM Group			
		Preceding interruption			Latter interruption			Latter interruption			Latter interruption			
Characteristic	Total	<1 d	≥1 d	*p*	<1 d	≥1 d	*p*	<4 d	≥4 d	*p*	Total	<4 d	≥4 d	*p*
	4510(100%)	1892(42.0%)	2618(58.0%)		1368(30.3%)	3142(69.7%)		3778(83.7%)	732(16.3%)		1452(100%)	727(50.0%)	727(50.0%)	
**Gender**				0.567			0.159			0.753				0.860
Male	3253(72.1%)	1356(30.1%)	1897(42.1%)		967(21.4%)	2286(50.7%)		2721(60.3%)	532(11.8%)		1058(72.8%)	531(36.5%)	527(36.2%)	
Female	1257(27.9%)	536(11.9%)	721(16.0%)		401(8.9%)	856(19.0%)		1057(23.4%)	200(4.4%)		396(27.2%)	196(3.50%)	200(13.8%)	
**Histology**				0.082			0.886			1.000				0.076
WHO Type I–II	58(1.3%)	31(0.7%)	27(0.6%)		18(0.4%)	40(0.9%)		49(1.1%)	9(0.2%)		21(1.4%)	15(1.0%)	6(0.4%)	
WHO Type III	4452(98.7%)	1861(41.3%)	2591(57.5%)		1350(29.9%)	3102(68.8%)		3729(82.7%)	723(16.0%)		1433(98.6%)	712(49.0%)	721(49.6%)	
**Age, year**					0.693		0.331			0.545				0.916
≤45	2357(52.3%)	1001(22.2%)	1356(30.1%)		730(16.2%)	1627(36.1%)		1982(43.9%)	375(8.3%)		739(50.8%)	368(25.3%)	371(25.5%)	
＞45	2153(47.7%)	891(19.8%)	1262(28.0%)		638(14.1%)	1515(33.6%)		1796(39.8%)	357(7.9%)		715(49.2%)	359(24.7%)	356(24.5%)	
**Smoking history**				0.183			0.022			0.309				0.551
No	2951(65.4%)	1259(27.9%)	1692(37.5%)		929(20.6%)	2022(44.8%)		2484(55.1%)	467(10.3%)		912(62.7%)	450(320.9%)	462(31.8%)	
Yes	1559(34.6%)	633(14.0%)	926(20.5%)		439(9.7%)	1120(24.8%)		1294(28.7%)	265(5.9%)		542(37.3%)	277(19.1%)	265(18.2%)	
**Drinking history**				0.333			0.148			0.128				1.000
No	3827(84.9%)	1594(35.3%)	2233(49.5%)		1177(26.1%)	2650(58.8%)		3192(70.8%)	635(14.1%)		1261(86.7%)	630(43.3%)	631(43.4%)	
Yes	683(15.1%)	298(6.6%)	385(8.5%)		191(4.2%)	492(10.9%)		586(13.0%)	97(2.2%)		193(13.3%)	97(6.7%)	96(6.6%)	
**Concomitant**				1.000			0.944			0.073				1.000
No	3127(69.3%)	1312(29.1%)	1815(40.2%)		950(21.1%)	2177(48.3%)		2640(58.5%)	487(10.8%)		967(66.6%)	484(33.3%)	483(33.2%)	
Yes	1383(30.7%)	580(12.9%)	803(17.8%)		418(9.3%)	965(21.4%)		1138(25.2%)	245(5.4%)		487(33.4%)	243(16.7%)	244(16.8%)	
**Family history**				0.244			1.000			0.336				0.442
No	3324(73.7%)	1377(30.5%)	1947(43.2%)		1008(22.4%)	2316(51.4%)		2795(62.0%)	529(11.7%)		1064(73.2%)	539(37.1%)	525(36.1%)	
Yes	1186(26.3%)	515(11.4%)	671(14.9%)		360(8.0%)	826(18.3%)		983(21.8%)	203(4.5%)		390(26.8%)	188(12.9%)	202(13.9%)	
T stage^a^				<0.001			<0.001			0.136				0.517
T1–2	1371(30.4%)	660(14.6%)	711(15.8%)		520(11.5%)	851(18.9%)		1166(25.9%)	205(4.5%)		396(27.2%)	192(13.2%)	204(14.0%)	
T3–4	3139(69.6%)	1232(27.3%)	1907(42.3%)		848(18.8%)	2291(50.8%)		2612(57.9%)	527(11.7%)		1058(72.8%)	535(36.8%)	523(36.0%)	
**N stage** ^a^				0.043			0.404			0.588				0.285
N0–1	2802(62.1%)	1208(26.8%)	1594(35.3%)		837(18.6%)	1965(43.6%)		2354(52.2%)	448(9.9%)		871(59.9%)	425(29.2%)	446(30.7%)	
N2–3	1708(37.9%)	694(15.2%)	1024(22.7%)		531(11.8%)	1177(26.1%)		1424(31.6%)	284(6.3%)		583(40.1%)	302(20.8%)	281(19.3%)	
**Overall stage** ^a^				<0.001			<0.001			0.481				0.245
I–II	908(20.1%)	440(9.8%)	468(10.4%)		331(7.3%)	577(12.8%)		768(17.0%)	140(3.1%)		260(17.9%)	132(8.3%)	139(9.6%)	
III–IVa	3602(79.9%)	1452(32.2%)	2150(47.7%)		1037(23.0%)	2565(56.9%)		3010(66.7%)	592(13.1%)		1194(82.1%)	606(41.7%)	588(40.4%)	
**Prescribed fractions**				<0.001			<0.001			<0.001				1.000
28–31	2140(47.5%)	1352(30.0%)	788(17.5%)		1117(24.8%)	1023(22.7%)		1907(42.3%)	233(5.2%)		466(32.0%)	233(16.0%)	233(16.0%)	
32–35	2370(52.5%)	540(12.0%)	1830(40.6%)		251(5.6%)	2119(47.0%)		1871(41.5%)	499(11.1%)		988(68.0%)	494(34.0%)	494(34.0%)	
**Chemotherapy**				0.128			0.027			0.793				0.281
No	477(10.6%)	216(4.8%)	261(5.8%)		166(3.7%)	311(6.9%)		402(8.9%)	75(1.7%)		137(9.4%)	62(4.3%)	75(5.2%)	
Yes	4033(89.4%)	1676(37.2%)	2357(52.3%)		1202(26.7%)	2831(62.8%)		3376(74.9%)	657(14.6%)		1317(90.6%)	665(45.7%)	652(45.8%)	

Abbreviation: WHO, World Health Organization.

^a^ According to the eighth edition of the American Joint Commission on Cancer staging system.

### 3.2 Prognostic value of interruptions or not

We selected a uniform threshold of 1 d (<1 d vs. ≥1 d) to categorize the cohort into four groups for survival analysis: (a) 659 patients without interruptions (preceding interruption duration <1 d and latter interruption duration <1 d); (b) 709 patients in the preceding interruption alone group (preceding interruption duration ≥1 d and latter interruption duration <1 d); (c) 1233 patients in the latter interruption alone group (preceding interruption duration <1 day and latter interruption duration ≥1 d); and (d) 1909 patients in the biphasic interruption group (preceding interruption duration ≥1 d and latter interruption duration ≥1 d).

**FIGURE 1:**
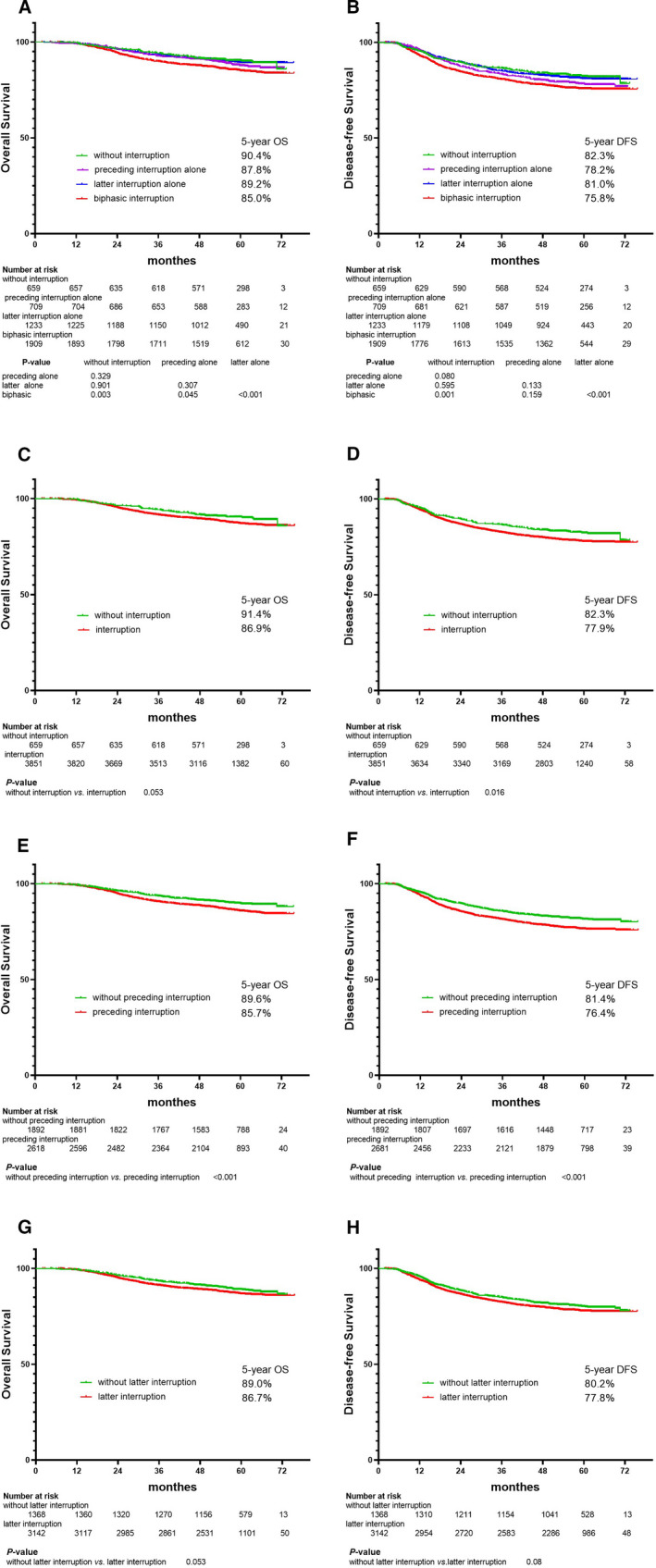
Kaplan–Meier's plots showed OS (A) and DFS (B) divided by preceding interruption (<1 d vs. ≥1 d) and latter interruption (<1 d vs. ≥1 d) into four groups; OS (C) and DFS (D) divided by preceding interruption (<1 d vs. ≥1 d) and latter interruption (<1 d vs. ≥1 d) into two groups; OS (E) and DFS (F) divided by preceding interruption (<1 d vs. ≥1 d) into 2 groups; OS (G) and DFS (H) divided by the latter interruption (<1 d vs. ≥1 d) into two groups, respectively

The OS and DFS curves for the patients without interruptions, in the preceding interruption alone group or in the latter interruption alone group, were indistinguishable with each other, and no significant differences were observed between them (*p* > 0.05 for all; Figure 1A,B), but the OS of the biphasic interruption group was significantly lower than other three groups (*p* < 0.05 for all; Figure 1A).

Although higher 5‐year OS was found in patients without interruptions (91.4% vs. 86.9%), no significant difference was observed (*p* = 0.053; Figure 1C). However, significant difference was found in the 5‐year DFS (82.3% vs. 77.9%, *p* = 0.016; Figure 1D).

Patients with preceding interruptions ≥1 d showed significantly lower OS and DFS rates than those without these interruptions (5‐year OS: 89.6% vs. 85.7%, *p* < 0.001; 5‐year DFS: 81.4% vs. 76.4%, *p* < 0.001, Figure 1E,F), whereas no significant survival differences was found between patients with and without latter interruptions ≥1 d (5‐year OS: 89.0% vs. 86.7%, *p* = 0.053; 5‐year DFS: 80.2% vs. 77.8%, *p* = 0.08, Figure 1G,H).

### 3.3 Prognostic value of interruptions with thresholds based on RPAS

The optimal threshold for preceding and latter interruptions, with respect to the OS, was 1 d and 4 d, based on RPAs. Therefore, patients were categorized into four groups: (a) 1513 patients in the without interruptions group (preceding interruption duration <1 d and latter interruption duration <4 d); (b) 2165 patients in the preceding interruption alone group (preceding interruption ≥1 d and latter interruption <4 d); (c) 279 patients in the latter interruption alone group (preceding interruption <1 d and latter interruption ≥4 d); and (d) 453 patients in the biphasic interruption group (preceding interruption ≥1 d and latter interruption ≥4 d).

**FIGURE 2:**
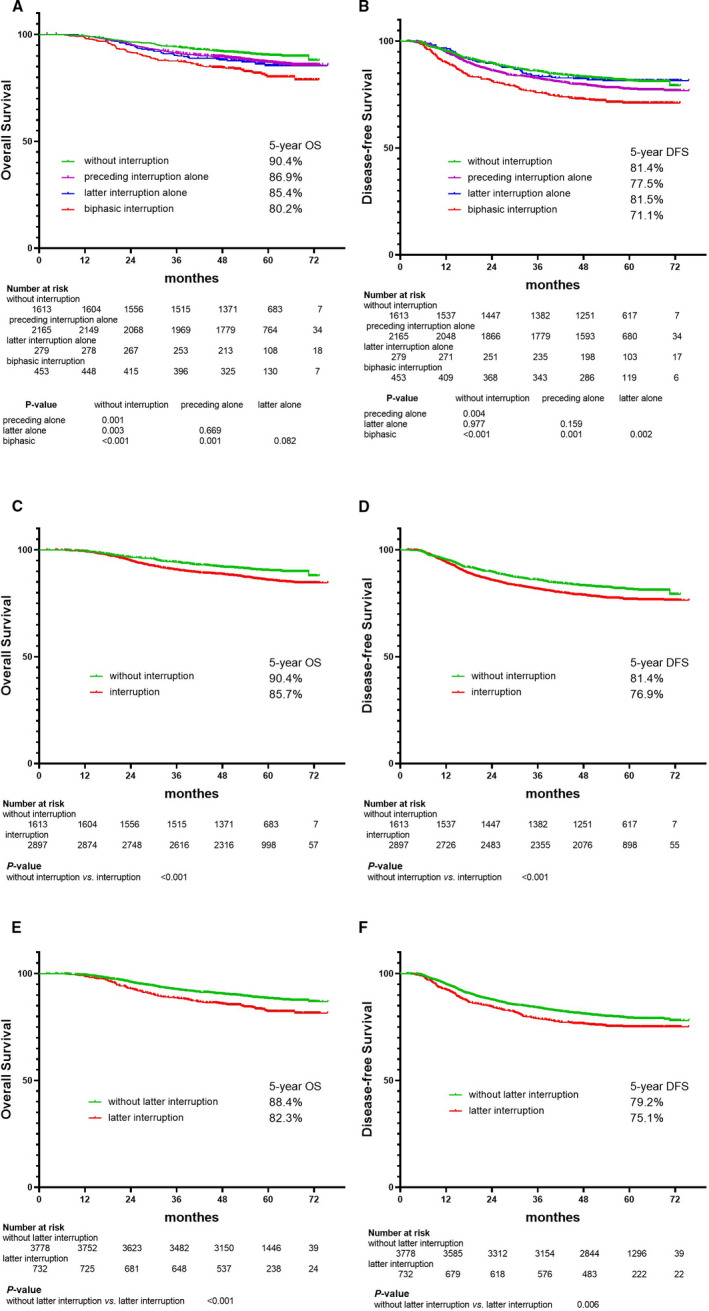
Kaplan–Meier's plots showed OS (A) and DFS (B) divided by the preceding interruption (<1 d vs. ≥1 d) and latter interruption (<4 d vs. ≥4 d) into four groups; OS (C) and DFS (D) divided by preceding interruption (<1 d vs. ≥1 d) and latter interruption (<4 d vs. ≥4 d) into two groups; OS (E) and DFS (F) divided by the latter interruption (<4 d vs. ≥4 d) into two groups, respectively

The OS and DFS curves for the preceding interruption alone group and latter interruption alone group were indistinguishable with each other, and no significant differences between them were observed (5‐year OS: 86.9% vs. 85.4%, *p* = 0.669; 5‐year DFS: 77.5% vs. 81.5%, *p* = 0.159). Patients with biphasic interruptions experienced significant detrimental effects on their OS and DFS than patients without interruption or with preceding interruption alone (*p* < 0.002 for all). Although patients with biphasic interruptions experienced significant detrimental effects on their DFS than latter interruption alone group (5‐year DFS: 71.1% vs. 81.5%, *p* = 0.002), no significant difference was found between biphasic interruption and latter interruption alone group (5‐year OS: 80.2% vs. 85.4%, *p* = 0.082) (Figure 2A,B).

Patients without interruptions had a significantly higher 5‐year OS (90.4% vs. 85.7%, *p* < 0.001; Figure 2C) and DFS (81.4% vs. 76.9%, *p* < 0.001; Figure 2D) compared with those in the interruption groups.

Compared to patients without interruptions, Patients with latter interruptions ≥4 d showed significantly inferior 5‐year OS (88.4% vs. 82.3%, *p* < 0.001; Figure 2 E and DFS (79.2% vs. 75.1%, *p* = 0.006; Figure 2F).

### 3.4 Times of interruptions in the univariate analyses and multivariate analyses

Consistent with survival analysis, preceding interruptions ≥1 d and latter interruptions ≥4 d were significant prognostic factors for OS and DFS (All *p* < 0.05) in the univariate analysis, but latter interruptions ≥1 d was not (*p* > 0.05, Table 2).

**TABLE 2:** Univariate analysis and Multivariate analyses of prognostic factors in 4510 NPC patients treated with IMRT

	Overall Survival				Disease‐free Survival			
Characteristic	Univariate		Multivariate		Univariate		Multivariate	
	HR (95% CI)	*p* value	HR (95% CI)	*p* value	HR (95% CI)	*p* value	HR (95% CI)	*p* value
Gender(male vs. female)	0.727(0.594–0.889)	0.002			0.707(0.607–0.825)	<0.001	0.790(0.667–0.937)	0.007
Histology (WHO type I–II vs. III)	0.639(0.351–1.160）	0.141			0.670(0.415–1.083)	0.102		
Age, year (≤45 vs. >45)	1.418(1.197–1.679）	<0.001	1.380(1.162–1.638)	<0.001	1.171(1.030–1.330)	0.015	1.160(1.020–1.320)	0.024
Smoking history (no vs. yes)	1.472(1.243–1.745)	<0.001	1.337(1.127–1.586)	0.001	1.361(1.195–1.549)	<0.001	1.169(1.012–1.355)	0.034
Drinking history (no vs. yes)	1.250(1.004–1.555)	0.046			1.084(0.911–1.290)	0.365		
Concomitant (no vs. yes)	1.321(1.109–1.573)	0.002	1.304(1.093–1.557)	0.003	1.137(0.993–1.303)	0.062		
Family history (no vs. yes)	0.844(0.692–1.029)	0.093			0.928(0.801–1.075)	0.319		
T stage^a^ (T1–2 vs. T3–4)	2.115(1.696–2.636)	<0.001	1.513(1.131–2.025)	0.005	1.860(1.585–2.182)	<0.001	1.325(1.064–1.649)	0.012
N stage^a^ (N0–1 vs. N2–3)	2.025(1.710–2.397)	<0.001	1.807(1.499–2.177)	<0.001	1.834(1.614–2.083)	<0.001	1.597(1.384–1.842)	<0.001
Overall stage^a^ (I–II vs. III–IVa)	3.426(2.473–4.745)	<0.001	1.645(1.052–2.574)	0.029	2.812(2.248–3.519)	<0.001	1.614(1.171–2.225)	0.003
Prescribed fractions (28–31 vs. 32–35)	1.642(1.379–1.955)	<0.001	1.297(1.070–1.573)	0.008	1.600(1.403–1.825)	<0.001	1.448(1.229–1.706)	<0.001
Chemotherapy (no vs. yes)	1.419(1.035–1.943)	0.03			1.645(1.281–2.112)	<0.001		
Preceding interruption (<1 d vs. ≥1 d)	1.404(1.176–1.675)	<0.001			1.315(1.152–1.502)	<0.001	1.425(1.024–1.982)	0.035
Latter interruption (<1 d vs. ≥1 d)	1.205(0.997–1.456)	0.053			1.133(0.984–1.306)	0.083		
Latter interruption (<4 d vs.≥ 4 d)	1.522(1.243–1.864)	<0.001	1.404(1.143–1.723)	0.001	1.252(1.064–1.473)	0.007	1.351(1.105–1.652)	0.003
Interruption (no vs. yes, 1–1 d)	1.290(0.996–1.672)	0.054			1.269(1.044–1.542)	0.017		
Interruption (no vs. yes, 1–4 d)	1.502(1.244–1.812)	<0.001			1.286(1.120–1.447)	<0.001		

*p* values were calculated using an adjusted Cox proportional hazards model.

Abbreviations: CI, confidence interval; HR, hazard ratio; IMRT, intensity modulated radiation therapy; WHO, World Health Organization.

^a^ According to the eighth edition of the American Joint Commission on Cancer staging system.

On multivariate analyses, latter interruptions ≥4 d was an independent unfavorable prognostic factor for OS (HR, 1.404; 95% CI, 1.143–1.723, *p* = 0.001) and DFS (HR, 1.351; 95% CI, 1.105–1.652; *p* = 0.003). Preceding interruptions ≥1 d was an independent factor in the poorer outcome for DFS (HR, 1.425; 95% CI, 1.024–1.982; *p* = 0.035) but not OS (Table 2).

### 3.5 Effect of chemotherapy on interruptions

**FIGURE 3:**
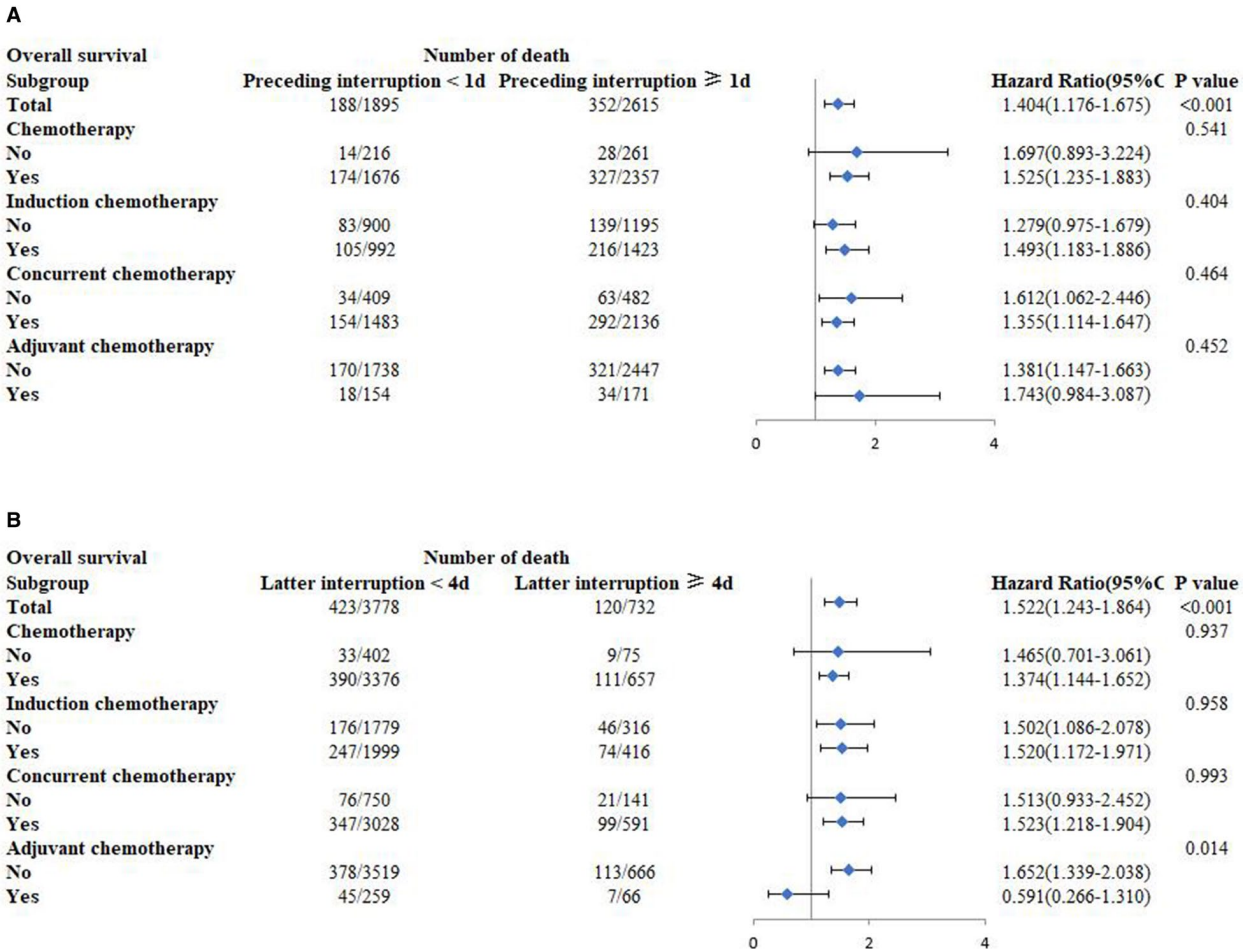
*3*Prognostic effects of: A, preceding interruption (<1 d vs. ≥1 d), B, latter interruption (<4 d vs. ≥4 d) on overall survival, stratified by chemotherapy characteristics in subgroups

Further subgroup analyses were conducted on OS to identify the value of chemotherapy on interruptions. We found that there were no interactions between the chemotherapy variables and preceding interruptions (Figure 3A). However, interactions of the latter interruptions (with a 4 d threshold) with AC were observed with respect to OS (*p* **‐**interaction = 0.014; Figure 3B).

**FIGURE 4:**
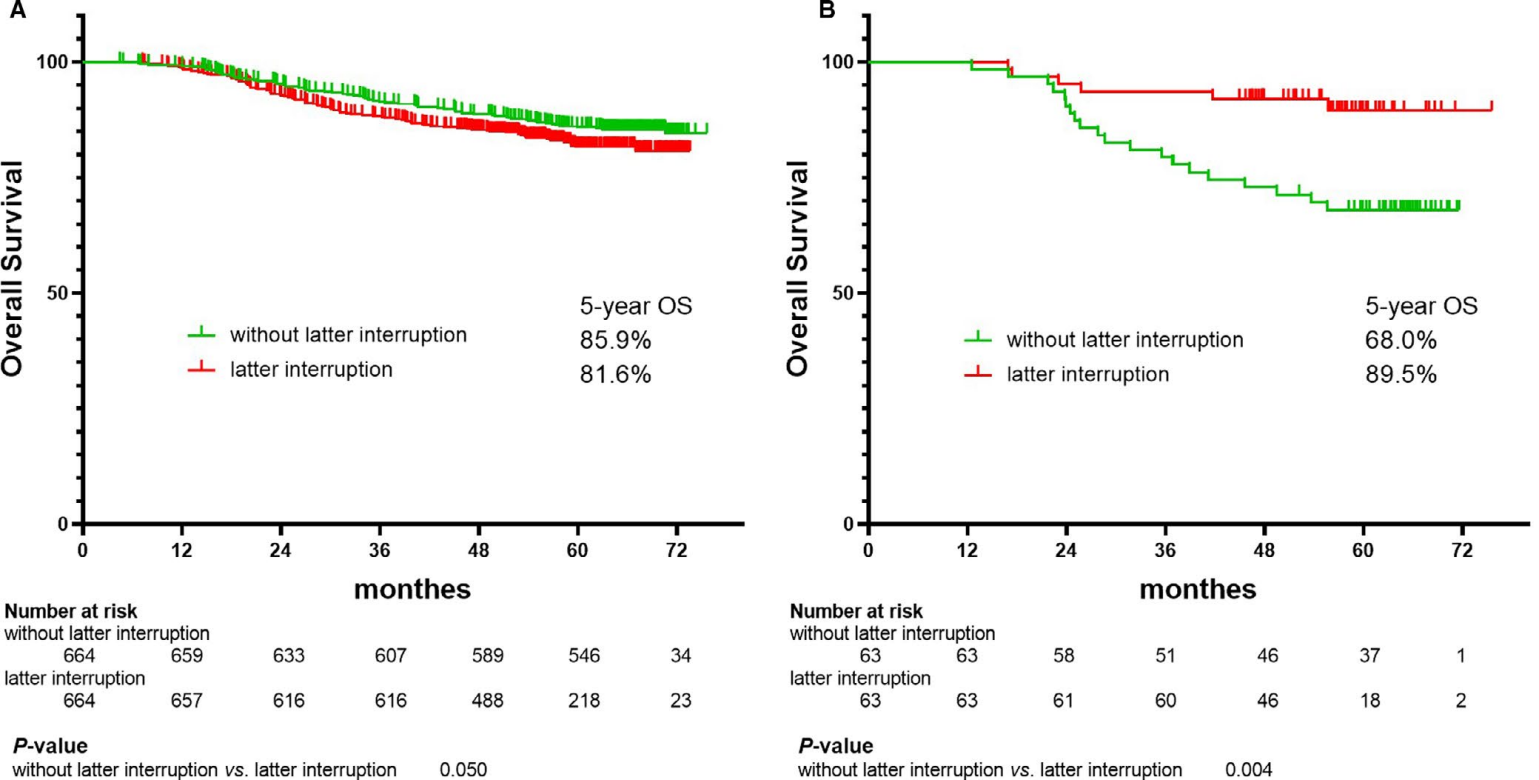
Kaplan–Meier's plots showed OS of latter interruption (<4 d vs. ≥4 d) in patients treated without (A) and with (B) AC

After adjusting for risk by PSM, patients without AC (Table 1), those with latter interruptions showed detrimental effects (using the 4 d threshold) on their 5‐year OS compared to those without latter interruptions (85.9% vs. 81.6%, *p* = 0.050; Figure 4A), while patients with AC, who experienced latter interruptions, showed better survival (68.0% vs. 89.5%, *p* = 0.003; Figure 4B).

## 4 DISCUSSION

This is the first study to quantify the prognostic impact of RT interruptions occurring at different times on patients with NPC treated by IMRT. Our results revealed the detrimental effects of interruptions, with respect to their time of occurrence, in which preceding interruptions ≥1 d and latter interruptions ≥4 d were associated with increased mortality. Further analysis suggests that AC might improve survival in patients with latter interruptions.

So far, the results of studies on the impact of interruptions during radiation treatment on the prognosis of NPC have been inconsistent. The studies conducted by Su et al, Li et al, and Stoker et al, suggested no significant adverse effects of treatment interruptions on survival.^10^, ^11^, ^12^ Yao et al. and Xu et al. observed a trend in the association of a poor prognosis with longer interruptions, but the number of days of interruption varied between their studies.^13^, ^14^, ^15^ Our findings confirmed the detrimental effect of interruptions on the survival of patients with NPC. Based on an analysis of nearly 8000 patients, Yao et al reported that interruptions should be limited to under 7 days to avoid the risk of an association with treatment failure.^15^ According to our results, no interruptions were encouraged during the early course of treatment, and interruptions were limited to under 4 d during the latter course. The suggested limits for interruptions during the entire course of radical IMRT were approximate because calculations of the planned radiation times were different in these two studies (Yao et al assumed a Monday start, while we used the actual date).

Few studies mentioned the times of treatment interruptions in patients with head and neck cancer receiving 2DRT era. In Danish split‐course trial, local control was found to decrease if a gap of 3 weeks was scheduled before the final 2.5 weeks of RT in patients with laryngocarcinoma and Pharyngeal cancer.^18^ Herrmann H and his colleagues found adverse effects of a break after the first 3 weeks of radiotherapy, with survival dropped to 18% to 25%, whereas no negative impact if the break occurred before.^19^ Nevertheless, another study reported that the local tumor control of patients with supraglottic larynx squamous cell carcinoma, whose gaps began during the middle period of radiotherapy (Days 20–29), was inappreciable from those who did not have interruption, and poorer local control were associated with interruptions that began in the other period of treatment.^20^

The timing of a single interruption (≥1 d) in patients with NPC, before or after the fourth week, had the same negative impact on local control.^6^ In our study, patients with interruptions occurring only in the preceding or latter periods shared comparable survival rates, which was consistent with studies of 2DRT. However, no detrimental effect of an interruption that occurred only one time was observed if the threshold was 1 d. The discrepancies between our study and previous studies were probably caused by the development of imagine technology, radiotherapy, chemotherapy,^21^ and other factors, such as varied inclusion criteria, population demographics, treatment strategies, and end points.

Patients with preceding interruptions ≥1 d exhibited a significant association with poorer survival. On the one hand, the accelerated repopulation of tumor cells during interruptions likely played an important role in this observation.^22^ On the other hand, exfoliated tumor cells caused by radiation were likely to diffuse through unblocked vessels and lymphatics during the preceding period of radiation. However, preceding interruptions failed to be an independent prognosis factor in our study. Radiation time may be shortened during the latter period intentionally by delivering more than five fractions a week^23^, ^24^ to counteract the effect of the preceding interruption.

In the multivariate analysis, interruption in latter, but not the entire period of radiation, was an independent adverse factor for OS. Interestingly, no significant difference was found in OS during latter interruptions of 1 d, while a latter interruption ≥4 d had significant detrimental effects on survival. Possible reasons for this “insensitivity” include the reduced tumor volume^25^ and blocked vessels and lymphatics during the latter period of radiation, which may have slowed the speed of spread. Latter interruptions ≥4 d induced an accelerated repopulation of tumor cells, generating micrometastasis. Meanwhile, accumulated treatment toxicity interfered with the delivery of subsequent chemotherapy,^26^ which was unable to eliminate the proliferation. The further analysis in our study suggests that AC significantly decrease the risk of deaths among patients with latter interruptions. For example, patients with high‐risk indicators before treatment^27^ and those with persistently detectable postradiation Epstein–Barr virus DNA both benefited from metronomic AC.^28^

We mainly focused on the impact of the times of interruptions and possibilities for salvaging its management, which would be helpful in clinical work. Nevertheless, this study had certain limitations. First, this was a retrospective study: the chemotherapy regimens were various, and patients with high risk of recurrence before treatment or residual tumor, detectable EBV DNA, persistent symptoms (e.g., headache) after radiotherapy were suggest to received AC, which mainly depend on their doctors, and might have resulted in selection bias. Hence, the result should be validated by further prospective studies. Second, whether the interruptions were continuous or discontinuous might have influenced differences in the effects. Finally, the study failed to mention treatment‐related toxicities because data on acute and late toxicities in the intelligence platform were lacking.

## 5 CONCLUSION

In conclusion, the latter interruption during RT was an independent prognostic factor in patients with NPC. Patients with preceding interruptions ≥1 d and/or latter interruptions ≥4 d were associated with poorer survival. As such, clinicians should make additional efforts to limit radiation interruptions.

## AUTHORS’ CONTRIBUTIONS

Conception and design: Yan‐ping Mao, Ying Sun, Xing‐li Yang; Financial support: Ying Sun, Jun Ma, Guan‐Qun Zhou; Administrative support: Guan‐Qun Zhou, Ying Sun, Yan‐ping Mao; Provision of study materials or patients: Yan‐ping Mao, Ying Sun, Xing‐li Yang; Collection and assembly of data: Xing‐Li Yang, Yan‐Ping Mao, Li Lin, Lu‐Lu Zhang, Fo‐Ping Chen, Jia‐Wei Lv, Jia Kou, Dan‐Wan Wen; Data analysis and interpretation: Xing‐Li Yang, (E‐mail: yangxingl@sysucc.org.cn); Manuscript writing: All authors; Final approval of manuscript: All authors.

## Supporting information

Supplementary Material

## Data Availability

This retrospective study was approved by the institutional ethics committee and the informed consent was granted a waiver. Key raw data were uploaded onto the Research Data Deposit public platform (RDD), with the approval RDD number of RDDA2020001485.
