# Supplementary material for: Prognostic value of radiation interruption in different periods for nasopharyngeal carcinoma patients in the intensity‐modulated radiation therapy era
Source: Cancer Med. 2020 Oct 27;10(1):143–55. doi: 10.1002/cam4.3580 (PMC7826475; doi:10.1002/cam4.3580)
Supplement: Supplementary file 1 — Supplementary Material [file CAM4-10-143-s001.docx]

**Supplementary materials**

**Table1. Correlation between concurrent chemotherapy regimens and interruptions**

| Concurrent chemotherapy | Cisplatin | Others | P value |
| --- | --- | --- | --- |
| Total | 2258 | 1361 |  |
| Without Preceding interruption (<1day) | 825（55.5%） | 661（44.5%） | <0.001 |
| With Preceding interruption (≥1day) | 1433（67.2%） | 700（32.8%） |  |
|  |  |  |  |
| Without Latter interruption (<1day) | 153(55.4%) | 123(44.6%) | 0.014 |
| With Latter interruption (≥1day) | 2105(63.0%) | 1238(37.0%) |  |
|  |  |  |  |
| Without Latter interruption (<4day) | 1454(61.5%) | 912(38.5%) | 0.113 |
| With Latter interruption (≥4day) | 804(64.2%) | 449(35.8%) |  |

**Table 2. Correlation between induction chemotherapy regimens and interruptions**

| Induction chemotherapy | TPF | GP/TP/PF | P value |
| --- | --- | --- | --- |
| Total | 1539 | 876 |  |
| Without Preceding interruption (<1day) | 667(43.3%) | 327(37.3%) | 0.004 |
| With Preceding interruption (≥1day) | 872(56.7%) | 549(67.2) |  |
|  |  |  |  |
| Without Latter interruption (<1day) | 126（65.6%） | 66（34.3%） | 0.585 |
| With Latter interruption (≥1day) | 1413（63.6%） | 810（36.4%） |  |
|  |  |  |  |
| Without Latter interruption (<4day) | 1002（62.9%） | 592（37.1%） | 0.228 |
| With Latter interruption (≥4day) | 537（65.4%） | 284（36.3%） |  |

Abbreviation: TPF, Cisplatin, 5-fluorouracil, and Docetaxel; GP, Gemcitabine and Cisplatin; TP, Docetaxel and Cisplatin; PF, Cisplatin with 5-fluorouracil.
